# Targeted Conservative Cointegrate Formation Mediated by IS*26* Family Members Requires Sequence Identity at the Reacting End

**DOI:** 10.1128/mSphere.01321-20

**Published:** 2021-01-27

**Authors:** Christopher J. Harmer, Ruth M. Hall

**Affiliations:** a School of Life and Environmental Sciences, The University of Sydney, NSW, Australia; University of Iowa

**Keywords:** cointegrate, Holliday junction, IS*26*, insertion sequence, mobile genetic element, strand transfer, transposase

## Abstract

IS*26* forms cointegrates using two distinct routes, a copy-in mechanism involving one insertion sequence (IS) and a target and a targeted conservative mechanism involving two ISs in different DNA molecules. In this study, the ability of IS*26* and some close relatives, IS*1006*, IS*1008*, and a natural hybrid, IS*1006/IS1008*, which are found predominantly in Acinetobacter spp., to interact was examined. IS*1006/1008* consists of 175 bp from IS*1006* at the left end, with the remainder from IS*1008.* These ISs all have the same 14-bp terminal inverted repeats, and the Tnp26, Tnp1006, and Tnp1008 transposases, with pairwise identities of 83.7% to 93.1%, should be able to recognize each other’s ends. In a *recA*-negative Escherichia coli strain, IS*1006*, IS*1008*, and IS*1006/1008* each formed cointegrates via the copy-in route and via the targeted conservative route, albeit at frequencies for the targeted reaction at least 10-fold lower than for IS*26*. However, using mixed pairs, targeted cointegration was detected only when IS*1008* was paired with the IS*1006*/*1008* hybrid, which also encodes Tnp1008, and the targeted cointegrates formed all arose from a reaction occurring at the end where the DNA sequences are identical. The reaction also occurred at the end with extended DNA identity using IS*26* paired with IS*26*::*catA1*, an artificially constructed IS*26* derivative that includes the *catA1* gene. Thus, both identical transposases and identical DNA sequences at the reacting end were required. These features indicate that the targeted conservative pathway proceeds via a single transposase-catalyzed strand transfer, followed by migration and resolution of the Holliday junction formed.

**IMPORTANCE** The IS*26* family includes the ISs that are commonly found associated with antibiotic resistance genes in multiply resistant Gram-negative and Gram-positive bacteria. IS*26* is most prevalent in Gram-negative species and can generate the clusters of antibiotic resistance genes interspersed with directly oriented IS*26* seen in multiply resistant pathogens. This ability relies on the novel dual mechanistic capabilities of IS*26* family members. However, the mechanism underlying the recently discovered targeted conservative mode of cointegrate formation mediated by IS*26*, IS*257/*IS*431*, and IS*1216*, which is unlike any previously studied IS movement mechanism, is not well understood. An important question is what features of the IS and the transposase are key to allowing IS*26* family members to undertake targeted conservative reaction. In this study, this question was addressed using mixed-partner crosses involving IS*26* and naturally occurring close relatives of IS*26* that are found near resistance genes in Acinetobacter baumannii and are widespread in Acinetobacter species.

## Abstract (precis)

The IS*26* family includes the ISs that are commonly found associated with antibiotic resistance genes in multiply resistant Gram-negative and Gram-positive bacteria. IS*26* is most prevalent in Gram-negative species and can generate the clusters of antibiotic resistance genes interspersed with directly oriented IS*26* seen in multiply resistant pathogens.

## INTRODUCTION

The IS*26* family, defined as the group of insertion sequences (IS) in the current IS*6* family that, according to a recent analysis, are clearly related to IS*26* (1), includes the IS that are most commonly found associated with antibiotic resistance genes in resistant Gram-negative (IS*26*) and Gram-positive (IS*257*/IS*431* and IS*1216*) bacteria (2). IS*26*, the best-studied member of this family, is known to form cointegrates using two mechanisms. A characteristic copy-in (previously called “replicative”) mechanism forms cointegrates between the DNA molecule containing IS*26* and a second target molecule (3, 4). This route duplicates the IS and the 8-bp target site, and the cointegrate, made up of the two participating molecules separated by directly oriented copies of IS*26* with the target site duplication surrounding the insertion, is the end product of the reaction. Apparent simple “transposition” products, namely, a single IS*26* surrounded by an 8-bp target site duplication (TSD) at a random site, are not known to be formed directly by IS*26*. However, they can arise at low frequency when the cointegrate is resolved by host-mediated homologous recombination between the two IS*26* copies in the cointegrate (reference 5 and references therein). The second transposase-dependent reaction was first recorded only recently and differs from the reactions described for any other IS studied to date. It forms cointegrates between two DNA molecules that both carry a copy of IS*26* (6, 7). The reaction occurs between the two IS*26* copies and is conservative, as the IS is not duplicated and a TSD is not generated. Cointegration via this route occurs at a frequency about 1,000-fold higher than via the copy-in route, making this the preferred reaction if two copies of IS*26*, each in a different molecule, are available (6, 8, 9). The product is identical to the one that would be formed by homologous recombination between the two IS copies, but the reaction occurs at a frequency over 1,000-fold higher than for homologous recombination, making it the preferred reaction even in a recombination-proficient host (7).

The shared characteristics of members of the IS*26* family, namely, related transposases and terminal inverted repeats (TIR), likely indicate an ability to perform the copy-in and targeted conservative cointegration reactions (1). Cointegrate formation via the copy-in route has been demonstrated for several IS*26* family members (see references in reference 1). That further IS can catalyze both reactions was recently verified using two IS*26* family members that are distantly related to IS*26*. IS*257* and IS*1216*, found in *Staphylococcus* spp. and *Enterococcus* spp., respectively, were able to form cointegrates by both the copy-in and the targeted conservative routes (10), as previously demonstrated for IS*26*. Granted the extent of the differences between the transposases of IS*26*, IS*257*, and IS*1216*, it was concluded that all members of the IS*26* family as recently defined (1) should be able to perform the same two reactions (10).

The targeted conservative reaction exhibits properties more akin to site-specific recombination than to transposition, and to date, little is known about the mechanism. The structure of the reaction products revealed that the IS are always in the same orientation in the cointegrate formed, indicating that the reaction occurs at like ends (either left with left or right with right) (8). By inactivating one or both of the outer ends of IS*26*, it was shown that the reaction between the two IS is not specific to one end but can occur at either end of the IS (8). Now, a key question is how the ends of the two participating IS are brought together productively. By analogy with other bacterial transposases, this is likely to be achieved via formation of a dimer, involving specific interactions between transposase molecules bound to different DNA sites involved in the reaction, leading to formation of a synaptic complex (11–14). One approach to identifying features of the transposase required for these interactions to occur involves the use of mutants that affect specific amino acids. However, we reasoned that interactions between closely related relatives of IS*26* could also be used to narrow down features that are essential for multimer formation.

A recent analysis of members of the IS*26* family identified IS*1006*, IS*1007*, and IS*1008* as members of the clade of the IS*26* family that includes those IS most closely related to IS*26* and found that the terminal 14 bp of their TIR are identical to the 14-bp IS*26* TIRs. The 14 bp are highly conserved in all members of the IS*26* family (1). These IS are found predominantly in Acinetobacter spp. and were first discovered in 2003 associated with heavy metal resistance determinants (15). IS*1008* was shown to form cointegrates, though the frequency of cointegrate formation was not reported. However, IS*1006*-mediated cointegrates were not detected (15). These IS have subsequently been found in several different Acinetobacter plasmids often associated with complex antibiotic resistance regions (16–19).

In this study, IS*1006*, IS*1008*, and an IS*1006-*IS*1008* hybrid designated IS*1006*/*1008* were first shown to perform both the untargeted copy-in reaction and the targeted conservative reaction. Then, to gain insight into the requirements for targeted conservative cointegrate formation, mixed pairs with IS*26* or with one another were tested for their ability to act together in this mode.

## RESULTS

### *IS1006*, *IS1008*, and IS*1006*/*1008*.

**FIG 1:**
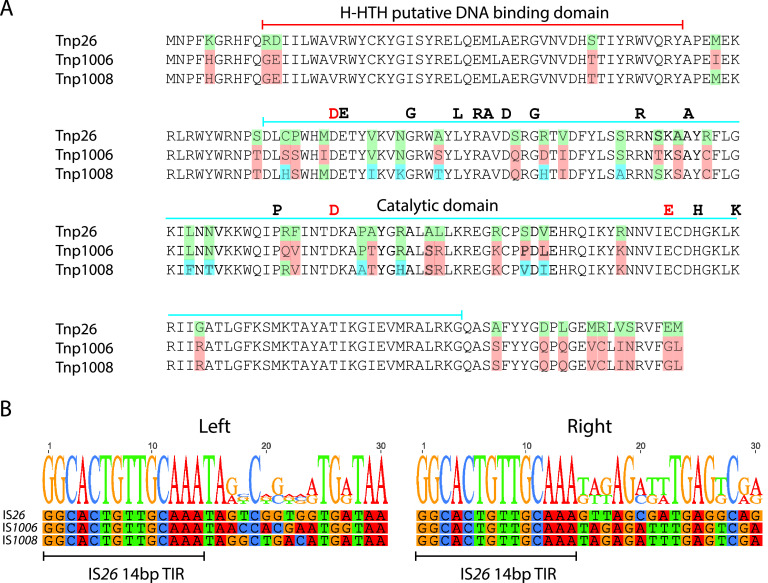
Comparison of IS*26*, IS*1006*, and IS*1008*. (A) Alignment of the amino acid sequences of the transposases of IS*26*, IS*1006*, and IS*1008*. Green, blue, and orange indicate amino acids that differ in one or more of the sequences. The extents of the H-HTH putative DNA binding domain and the DDE catalytic domain are marked above the sequences. The completely conserved DDE residues are marked by red letters, and residues conserved in >90% of the broader IS*26* family (1) are marked by black letters. The K180 residue, which is an R in one clade of the IS*26* family (1), is also marked. (B) Alignment of 30 bp of sequence at the left and right ends of IS*26*, IS*1006*, and IS*1008*. The sequences of the right ends have been reversed and complemented for ease of comparison. The 14-bp terminal inverted repeats (TIR) are marked by bars.

IS*26* shares 75.4% nucleotide identity with IS*1006* and 72.2% nucleotide identity with IS*1008*, and IS*1006* and IS*1008* share 87.4% nucleotide identity. The IS*26* transposase Tnp26 shares 85.2% and 83.7% amino acid identity with Tnp1008 and Tnp1006, respectively (Fig. 1A), and Tnp1008 and Tnp1006 are even more closely related, sharing 93.1% amino acid identity. The majority of the amino acid differences occur in the DDE catalytic domain (Fig. 1A). There are no differences between Tnp1006 and Tnp1008 in the predicted helix-helix-turn-helix (H-HTH) DNA binding domain and only three differences relative to Tnp26 in this domain (Fig. 1A).

**FIG 2:**
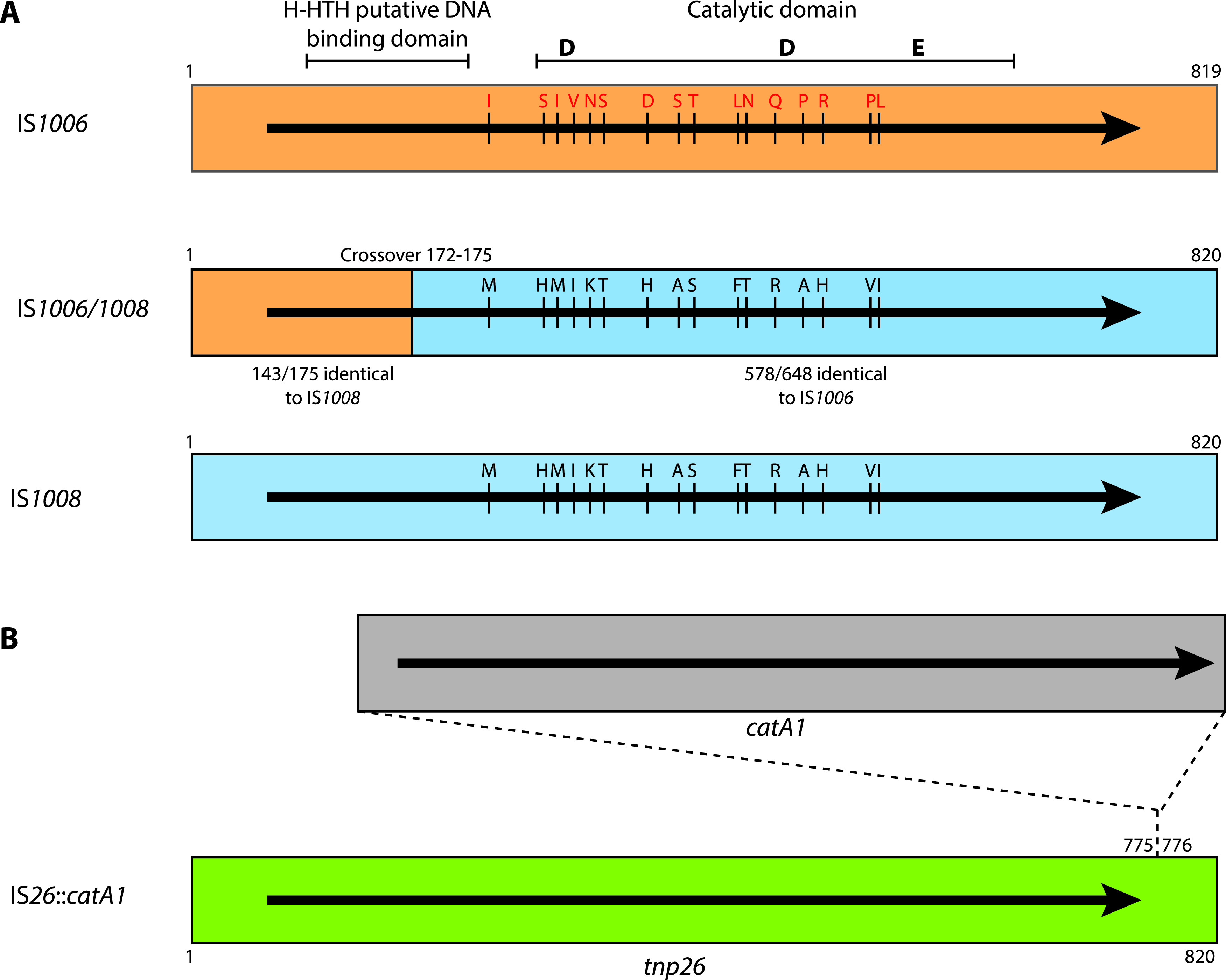
Schematic representations of IS*1006*, IS*1008*, the hybrid IS*1006*/*1008*, and IS*26*::*catA1*. (A) Nucleotide sequences belonging to IS*1006* and IS*1008* are shaded in orange and blue, respectively. The crossover between the two sequences in IS*1006*/*1008* is marked by a vertical line, indicating the first base belonging exclusively to IS*1008*. The extent and orientation of the transposase open reading frame are indicated by a black arrow. Amino acids that differ between the transposases are marked by red and black letters. The H-HTH putative DNA binding domain and the DDE catalytic domain are marked at the top. The positions of the conserved DDE catalytic triad are marked by bold letters. In IS*1006*/*1008*, the nucleotide identity between IS*1006* and IS*1008* is indicated in the two segments. Drawn to scale from GenBank accession numbers CP012956, CP041590, and KU744946 for IS*1006*, IS*1006*/*1008*, and IS*1008*, respectively. (B) The synthetic IS*26*::*catA1* construct is shown with IS*26* in green and the *catA1* gene fragment in orange. The extent and orientation of the *tnp26* and *catA1* genes are indicated by a black arrow. The position of the inserted *catA1* fragment in IS*26* is marked.

We have also identified an additional related IS, originally named IS*1008*-like (16) but henceforth called IS*1006*/*1008*, that is a hybrid formed between IS*1006* and IS*1008* (bases 42337 to 43155 in Acinetobacter baumannii strain J9 plasmid pJ9-3 [GenBank accession number CP041590]). The crossover occurs between bases 172 and 175, with the first 175 bases identical to IS*1006* and the remaining bases identical to IS*1008* (Fig. 2). The transposase encoded by IS*1006/1008* is identical to the IS*1008* transposase, as the nucleotide differences before the crossover do not result in any amino acid substitutions.

Importantly, the 16-bp terminal inverted repeats (TIR) of IS*1006*, IS*1008*, and IS*1006*/*1008* are identical to one another and to the IS*26* sequence at the left end. At the right end, the first 14 bp are identical to the 14-bp TIR of IS*26* (Fig. 1B). In the case of IS*1006* and IS*1008*, identity at their right ends extends beyond the 16-bp TIR for a total of 33 bp. As transposases generally recognize and bind to the TIR, we predict that the transposases of IS*26*, IS*1006*, IS*1008*, and IS*1006*/*1008* should be able to recognize each other’s TIR, opening the possibility that these related transposases can function together to perform the targeted conservative reaction.

### IS*1006*, IS*1008*, and IS*1006*/*1008* are active in copy-in and targeted conservative cointegrate formation.

Untargeted cointegrate formation in a *recA*-negative E. coli was examined using a mating-out assay to detect cointegrates formed between the conjugative plasmid R388 (Tp^r^) and the small, nonconjugative, nonmobilizable pUC19 plasmid containing IS*1006* (pRMH1011; Ap^r^), IS*1008* (pRMH1012; Ap^r^), or IS*1006*/*1008* (pRMH1013; Ap^r^) (Table 1). The three IS formed cointegrates at frequencies of 3.4 × 10^−7^ to 6.9 × 10^−7^ per transconjugant, averaged from three independent experiments (Table 2). These values are similar to the frequency for the reaction between IS*26* and R388 obtained in this study (5.1 × 10^−7^ cointegrates per transconjugant [Table 2]) and in previous studies (6, 8, 10).

**TABLE 1:** Plasmids used in this study

Plasmid	Description	Resistance phenotype^*a*^	Reference
pRMH977	IS*26* in pUC19	Ap	6
pRMH1011	IS*1006* in pUC19^*b*^^,^^*c*^	Ap	This study
pRMH1012	IS*1008* in pUC19^*c*^^,^^*d*^	Ap	This study
pRMH1013	IS*1006*/*1008* in pUC19^*c*^^,^^*e*^	Ap	This study
pRMH1015	IS*26* with internal *catA1* gene in pUC19^*c*^^,^^*f*^	Ap Cm	C. J. Harmer and R. M. Hall, submitted for publication
pRMH762	IS*26* in pUC19	Ap	6
pRMH962	IS*26*-FS-L in pUC19	Ap	6
R388	IncW plasmid	Su Tp	22
R388::IS*26*	IS*26* in R388	Su Tp	6
R388::IS*26*-2	IS*26* in R388^*g*^	Su Tp	This study
R388::IS*26*-FS-R	R388::IS*26* frameshift mutant	Su Tp	6
R388::IS*1006*	IS*1006* in R388^*b*^^,^^*g*^	Su Tp	This study
R388::IS*1008*	IS*1008* in R388^*d*^^,^^*g*^	Su Tp	This study
R388::IS*1006*/*1008*	IS*1006*/*1008* in R388^*e*^^,^^*g*^	Su Tp	This study

a Ap, ampicillin; Su, sulfamethoxazole; Tp, trimethoprim.

b Bases 39531 to 40583 from pD36-4 (GenBank accession no. CP012956).

c Insert cloned into the pUC19 BamHI site by Gibson assembly.

d Bases 88655 to 90375 from pA297-3 (GenBank accession no. KU744946).

e Bases 42241 to 43233 from pJ9-3 (GenBank accession no. CP041590).

f Synthetic construct, equivalent to pRMH977, with bases 133211 to 133909 of pRMH760 (GenBank accession no. KF976462) containing the *catA1* gene and its natural promoter inserted between bases 775 and 776 of IS*26*.

g Insert cloned between the two R388 HindIII sites by Gibson assembly.

**TABLE 2:** Frequency of cointegrate formation

IS^*b*^	Target	Cointegration frequency^*a*^	
		Range	Mean (SD)
Untargeted copy-in			
IS*26*	R388	4.8 × 10^−7^ to 5.4 × 10^−7^	5.1 × 10^−7^ (2.9 × 10^−8^)
IS*1006*	R388	3.2 × 10^−7^ to 8.0 × 10^−7^	5.9 × 10^−7^ (2.5 × 10^−7^)
IS*1008*	R388	1.0 × 10^−7^ to 4.7 × 10^−7^	3.4 × 10^−7^ (2.1 × 10^−7^)
IS*1006*/*1008*	R388	5.8 × 10^−7^ to 8.7 × 10^−7^	6.8 × 10^−7^ (1.7 × 10^−7^)
Targeted conservative			
IS*26*	R388::IS*26*	4.6 × 10^−4^ to 7.2 × 10^−4^	6.1 × 10^−4^ (1.4 × 10^−4^)
IS*26*	R388::IS*26*-2	4.7 × 10^−4^ to 9.7 × 10^−4^	8.0 × 10^−4^ (2.9 × 10^−4^)
IS*1006*	R388::IS*1006*	2.4 × 10^−5^ to 7.7 × 10^−5^	5.1 × 10^−5^ (2.4 × 10^−5^)
IS*1008*	R388::IS*1008*	1.0 × 10^−5^ to 5.1 × 10^−5^	3.1 × 10^−5^ (2.1 × 10^−5^)
IS*1006*/*1008*	R388:: IS*1006*/*1008*	3.0 × 10^−5^ to 7.8 × 10^−5^	4.7 × 10^−5^ (2.7 × 10^−5^)

a Frequency measured as cointegrates per transconjugant. Three independent determinations were made per experiment.

b IS in the pUC19 backbone.

To confirm that target selection was not sequence specific, the locations of the junctions between the IS*1006-*, IS*1008*-, and IS*1006/1008*-containing plasmids and R388 in the cointegrate were initially determined using the restriction mapping strategy with BglII and BsiWI described in Materials and Methods. Analysis of the fragments showed that in each cointegrate examined, pRMH1011, pRM1012, or pRMH1013 had incorporated at a different position in the R388 backbone (see Fig. S1 in the supplemental material). Then, a series of PCR primers was designed (Table S1) to map and sequence the precise boundaries and relative orientation of the two plasmids in six cointegrates from each experiment. In each case, both potential orientations of the plasmids relative to one another were observed (1 and 2 in Fig. S1).

10.1128/mSphere.01321-20.1 FIG S1 Cointegrate formation between pRMH1011 (IS*1006*), pRMH1012 (IS*1008*), or pRMH1013 (IS*1006/1008*) and R388. The R388 backbone is drawn to scale from GenBank accession no. BR000038 with key resistance genes, genes involved in replication (*repA*), and genes involved in conjugative transfer (*tra*) shown as arrows inside the circular backbone. Colored arrows pointing towards the circular backbone indicate the location of the mapped cointegrates as follows: red, IS*1006*; blue, IS*1008*; and purple, IS*1006/1008*. 1, the cointegrate was oriented with the *tnp* genes in the same orientation as the R388 *repA* gene; 2, the opposite orientation. Download FIG S1, EPS file, 1.3 MB. Copyright © 2021 Harmer and Hall. 2021 Harmer and Hall. This content is distributed under the terms of the Creative Commons Attribution 4.0 International license.

10.1128/mSphere.01321-20.2 TABLE S1 Primers used in this study. Download Table S1, DOCX file, 0.02 MB. Copyright © 2021 Harmer and Hall. 2021 Harmer and Hall. This content is distributed under the terms of the Creative Commons Attribution 4.0 International license.

Targeted cointegration was tested using pRMH1011 (IS*1006* Ap^r^) and R388::IS*1006* (Tp^r^), pRMH1012 (IS*1008* Ap^r^) and R388::IS*1008* (Tp^r^), or pRMH1013 (IS*1006*/*1008* Ap^r^) and R388::IS*1006*/*1008* in a *recA*-negative background to ensure that all events detected were catalyzed by the available transposase. Cointegrates were formed between identical pairs of IS at frequencies ranging from 3.1 × 10^−5^ to 5.1 × 10^−5^ per transconjugant, averaged from three independent determinations (Table 2). These frequencies are approximately 100-fold higher than for the copy-in values. However, they are approximately 10-fold lower than the values obtained in this study (Table 2) and previously for the reaction between two wild-type IS*26* under the same conditions (5, 6, 8, 9), which average 4.3 × 10^−4^ cointegrates/transconjugant from 16 independent determinations.

The IS in R388::IS*1006*, R388::IS*1008*, and R388::IS*1006*/*1008* are located in a different backbone position in R388 from the IS*26* in R388::IS*26*, which has been used in all our previous experiments for IS*26* (6, 8, 10). To ensure that the backbone location did not affect the cointegration frequency, a new construct (R388::IS*26*-2) was made with the IS*26* cloned into the same location as IS*1006*, IS*1008*, and IS*1006*/*1008*. R388::IS*26*-2 formed targeted cointegrates with pRMH977 (IS*26*) at an average frequency of 8.0 × 10^−4^ per transconjugant, consistent with the value obtained for R388::IS*26* (Table 2).

**FIG 3:**
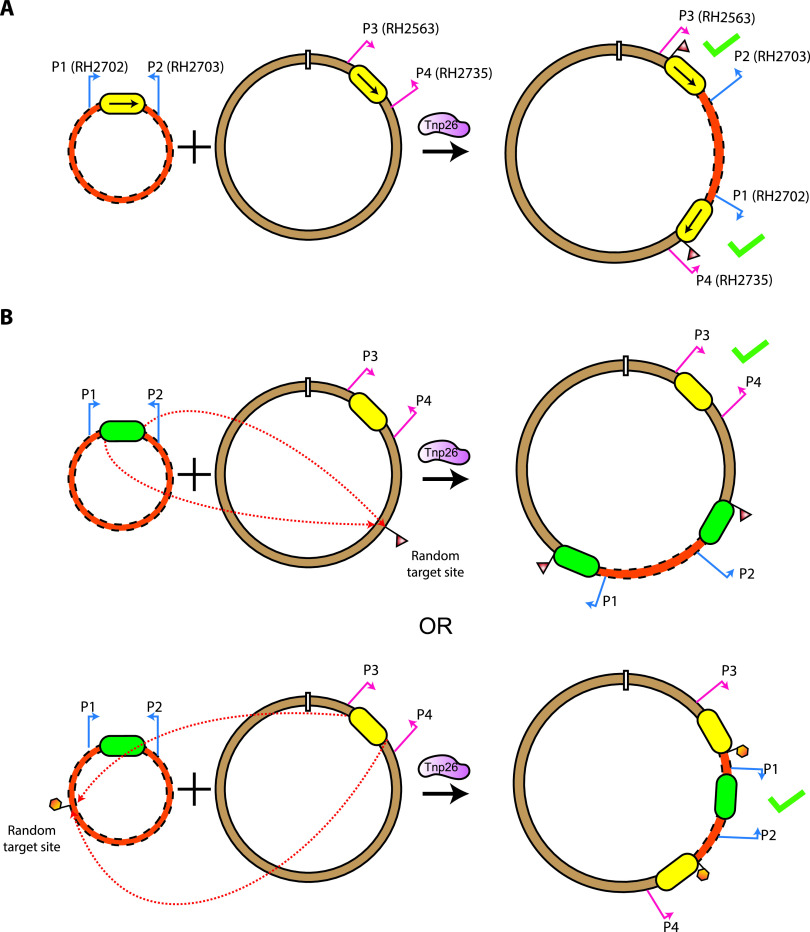
Cointegrate PCR mapping strategies. (A) Targeted conservative cointegrate formation. The two molecules containing the IS are indicated by a dashed orange line, and a solid brown line, with the IS shown as a yellow box. Primer pairs are denoted by bent arrows and labeled in their original molecule and in the cointegrate. A green check denotes the combination of primers that generate a positive PCR product from targeted cointegrates. (B) Strategy for determining the IS participating in the untargeted copy-in reaction. The molecules containing different IS*26* family members are denoted by a dashed orange line, and a solid brown line, with one IS indicated by a green box and the other by a yellow box. Primer pairs that amplify across each of the IS in their original position are indicated by bent arrows: P1 and P2 (blue arrows) and P3 and P4 (red arrows). A random target site and subsequent target site duplication (TSD) are indicated by triangular or hexagonal flags. Primer pairs that would generate an amplicon after cointegrate formation by each IS are indicated by a green tick.

Ten Ap^r^ Tp^r^ cointegrates from each of the three independent experiments for IS*1006*, IS*1008*, and IS*1006*/*1008* were screened by PCR using primer pairs (RH2563 with RH2703 and RH2702 with RH2735) that amplify across each of the two IS separating the two replicons in targeted cointegrates when the two IS in are the same orientation (Fig. 3A). This confirmed that in all instances pRMH1011, pRMH1012, or pRMH1013 had been incorporated adjacent to the existing IS in R388::IS*1006*, R388::IS*1008*, or R388::IS*1006*/*1008*, respectively. Hence, the IS*26* relatives IS*1006*, IS*1008*, and IS*1006*/*1008* are able to perform the targeted conservative cointegrate formation reaction previously demonstrated for IS*26*, IS*257*, and IS*1216*.

### Is cross-recognition possible between related IS*26* family members?

Granted their identical TIR and the high similarity between their transposases, the possibility that IS*1006*, IS*1008*, and/or IS*1006*/*1008* may be able to perform the targeted conservative reaction with IS*26* was examined. Both combinations where one molecule contained IS*1006*, IS*1008*, or IS*1006*/*1008* and the other molecule contained IS*26* were tested. E. coli UB5201 (Nx^r^) containing R388::IS*26* (Tp^r^) and either pRMH1011 (IS*1006* Ap^r^), pRMH1012 (IS*1008* Ap^r^), or pRMH1013 (IS*1006*/*1008* Ap^r^) were mated with E. coli UB1637 (Sm^r^), and in all three cases, Ap^r^ Tp^r^ cointegrates were detected (Table 3). However, the frequency of cointegrate formation, ranging from 3.7 × 10^−7^ to 9.1 × 10^−7^ cointegrates per transconjugant (Table 3), was significantly lower than that expected if the cointegrates were being formed via the targeted conservative route but similar to those obtained for the copy-in route (Table 2). In the reciprocal experiment, performed using E. coli UB5201 containing R388::IS*1006*, R388::IS*1008*, or R388::IS1006/*1008* and pRMH977 (IS*26* Ap^r^) as the donor, cointegrates formed at average frequencies of between 6.6 × 10^−7^ and 8.1 × 10^−7^ per transconjugant (Table 3). Again, these frequencies are similar to those obtained via the untargeted copy-in route for each IS (Table 2).

**TABLE 3:** Frequency of cointegrate formation between mixed pairs of IS

IS^*b*^	Target	Cointegration frequency^*a*^	
		Range	Mean (SD)
IS*26*	R388::IS*1006*	2.1 × 10^−7^ to 1.7 × 10^−6^	8.1 × 10^−7^ (7.6 × 10^−7^)
IS*26*	R388::IS*1008*	4.6 × 10^−7^ to 6.9 × 10^−7^	6.6 × 10^−7^ (3.3 × 10^−7^)
IS*26*	R388:: IS*1006*/*1008*	6.6 × 10^−7^ to 8.9 × 10^−7^	7.6 × 10^−7^ (1.2 × 10^−7^)
IS*1006*	R388::IS*26*	6.9 × 10^−7^ to 1.3 × 10^−6^	9.9 × 10^−7^ (3.1 × 10^−7^)
IS*1006*	R388::IS*1008*	1.5 × 10^−7^ to 6.0 × 10^−7^	4.0 × 10^−7^ (2.3 × 10^−7^)
IS*1006*	R388:: IS*1006*/*1008*	5.9 × 10^−7^ to 8.4 × 10^−7^	6.9 × 10^−7^ (1.3 × 10^−7^)
IS*1008*	R388::IS*26*	2.2 × 10^−7^ to 1.7 × 10^−6^	3.7 × 10^−7^ (1.8 × 10^−7^)
IS*1008*	R388::IS*1006*	5.2 × 10^−7^ to 6.9 × 10^−7^	5.9 × 10^−7^ (9.1 × 10^−8^)
IS*1008*^*c*^	R388:: IS*1006*/*1008*	4.5 × 10^−5^ to 6.7 × 10^−5^	5.6 × 10^−5^ (1.1 × 10^−5^)
IS*1006*/*1008*	R388::IS*26*	5.7 × 10^−7^ to 1.4 × 10^−6^	9.0 × 10^−7^ (4.7 × 10^−7^)
IS*1006*/*1008*	R388::IS*1006*	1.4 × 10^−7^ to 8.2 × 10^−7^	5.6 × 10^−7^ (3.7 × 10^−7^)
IS*1006*/*1008*^*c*^	R388::IS*1008*	2.5 × 10^−5^ to 5.4 × 10^−5^	4.2 × 10^−5^ (1.5 × 10^−5^)
IS*26*::*catA1*^*c*^	R388::IS*26*	3.2 × 10^−4^ to 7.6 × 10^−4^	5.5 × 10^−4^ (2.2 × 10^−4^)

a Frequency measured as cointegrates per transconjugant. Three independent determinations were made per experiment.

b IS in the pUC19 backbone.

c Shading indicates frequencies that are consistent with the targeted conservative reaction.

PCR screening of DNA from three Ap^r^ Tp^r^ cointegrates from each experiment (9 cointegrates per combination) did not generate the products expected if the reaction was targeted (Table 4). This indicates that the cointegrates that were detected had not formed via a targeted reaction between the mixed pairs of IS and had likely been formed by one of the IS present using the copy-in cointegration route at a random target site (Fig. 3B). To determine which IS formed the cointegrate, pairs of primers in the plasmid backbones that amplify across the original single IS were used to identify which IS remained in its original position, indicating that it had not mediated the cointegration event. As shown in Fig. 3B, primer P1 with P2 detects the IS in the pUC19 constructs (pRMH977, pRMH1011, pRMH1012, and pRMH1013), and primer P3 with P4 detects the IS in the R388 constructs (R388::IS*26*, R388::IS*1006*, R388::IS*1008*, and R388::IS*1006*/*1008*). DNA from the nine cointegrates from each IS combination screened as described above was examined using this strategy, and either the IS in pUC19 or the IS in R388 was still in the original position in each of the cointegrates tested (Table 4). Cointegrates were formed at approximately equal frequencies by the IS in pUC19 and the IS in R388, despite the difference in copy numbers of the two plasmids. Hence, the cointegrates had all been formed via the copy-in route and IS*1006*, IS*1008*, and IS*1006*/*1008* were not able to combine with IS*26* to perform the targeted conservative reaction.

**TABLE 4:** PCR screening of cointegrates formed between mixed reactions of molecules containing IS*26*, IS*1006*, IS*1008*, or IS*1006*/*1008*

IS in:		No. of cointegrates tested	Screening PCR^*a*^		
R388	pUC19		Targeted reaction	R388 is original	pUC19 is original
IS*26*	IS*1006*	9	0	2	7
IS*26*	IS*1008*	9	0	4	5
IS*26*	IS*1006*/*1008*	9	0	6	3
IS*1006*	IS*26*	9	0	3	6
IS*1006*	IS*1008*	9	0	6	3
IS*1006*	IS*1006*/*1008*	9	0	5	4
IS*1008*	IS*26*	9	0	5	4
IS*1008*	IS*1006*	9	0	4	5
IS*1008*	IS*1006*/*1008*	15	15	—	—
IS*1006*/*1008*	IS*26*	9	0	3	6
IS*1006*/*1008*	IS*1006*	9	0	4	5
IS*1006*/*1008*	IS*1008*	15	15	—	—
IS*26*	IS*26*::*catA1*	30	30	—	—

a —, not tested.

### Can IS*1006* and IS*1008* act together?

Even though IS*1006* and IS*1008* were unable to react with IS*26*, it remained possible that they may be able to react with one another, as Tnp1006 and Tnp1008 share 93.1% amino acid identity and 96.6% amino acid similarity (226/234 residues), with no differences in the putative H-HTH DNA binding domain, and they have identical 16-bp TIR. However, Ap^r^ Tp^r^ cointegrates that formed between R388::IS*1006* and pRMH1012 (IS*1008*) or between R388::IS*1008* and pRMH1011 (IS*1006*) were detected at mean frequencies of 5.9 × 10^−7^ or 4.0 × 10^−7^ per transconjugant (averaged from three independent determinations) (Table 3), suggesting the copy-in route. Using the PCR screening strategy illustrated in Fig. 3B with DNA from three cointegrates from each independent cross (18 in total), it was shown that none had been formed by the targeted reaction. Rather, all 18 had been formed by either IS*1006* or IS*1008* performing an untargeted copy-in reaction at a naive site in the second molecule (Table 4), and again, no preference for either IS was observed. Hence, despite their close relationship, IS*1006* and IS*1008* were unable to act together.

### IS*1008* and IS*1006*/*1008* can act together.

IS*1008* and IS*1006/1008* both encode Tnp1008, and they share 649 bp of nucleotide sequence identity at their right ends (Fig. 2). When pRMH1013 (IS*1006/1008*) was tested in combination with R388::IS*1008*, cointegrates formed at an average frequency of 4.2 × 10^−5^ per transconjugant and the reciprocal experiment, pRMH1012 (IS*1008*) in combination with R388::IS*1006/1008*, yielded a similar result (shaded rows in Table 3). These values are similar to the frequencies observed for the targeted cointegration reactions between identical IS (Table 2). PCR screening of a total of 30 cointegrates (5 Ap^r^ Tp^r^ cointegrates from each of the three independent experiments per pair) confirmed that in all instances, pRMH1012 or pRMH1013 had been incorporated adjacent to the existing IS in R388::IS*1006/1008* or R388::IS*1008*, with the two IS in the same orientation. Hence, IS*1006/1008* and IS*1008* are able to act together in the targeted conservative cointegration reaction.

### The targeted cointegration reaction requires extended identical DNA sequences.

**FIG 4:**
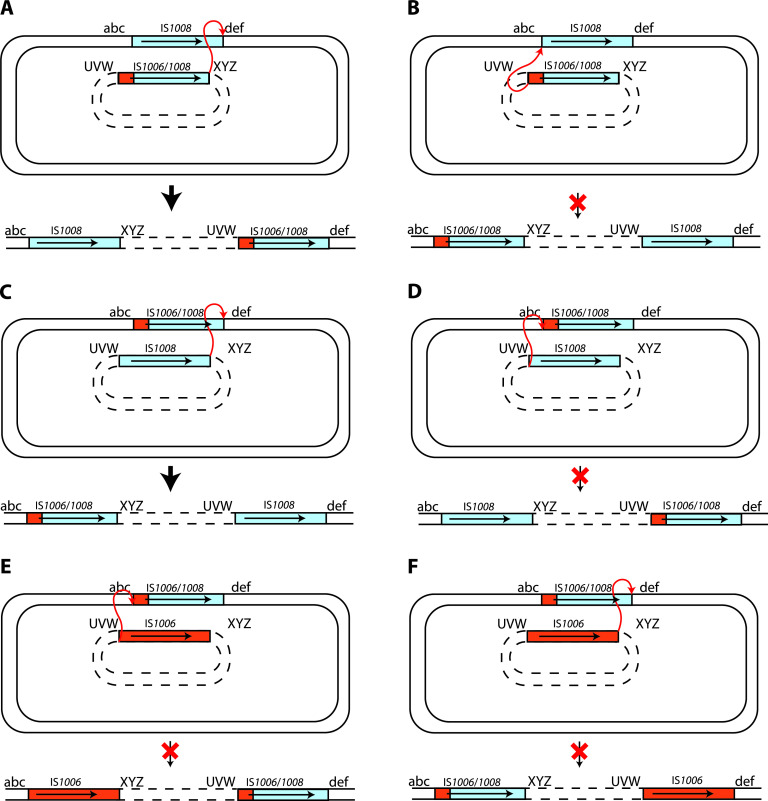
Outcome of targeted conservative cointegrate formation between IS*1006*/IS*1008* and IS*1008* or IS*1006*. Shown are reactions occurring between the right ends (A and C) and the left ends (B and D) of IS*1008* and IS*1006*/*1008* or between the left (E) or right (F) ends of IS*1006* and IS*1006*/*1008*. The predicted cointegrate configurations are depicted below the vertical arrow. The red cross indicates where a viable cointegrate was not detected. IS*1008-* and IS*1006*-derived sequences are in blue and orange, respectively. An arrow indicates the position and orientation of the transposase gene. The two DNA molecules are identified by either solid lines (R388) or dashed lines (pUC19 derivatives). For clarity, the specific sequences next to each IS are marked with abc, def, UVW, or XYZ. The reacting ends are indicated by a red double-ended arrow.

We considered the possibility that nucleotide identity could be critical if branch migration is needed to process the strand transfer intermediate, predicted to be a nicked Holliday junction (HJ) formed via a single-strand transfer event, formed by the transposase. Hence, we examined whether the reaction had occurred only (or preferentially) at the right end, where IS*1008* and IS*1006/1008* share significant length of nucleotide identity (649 bp), and not at the left end, where only the first 16 bp are identical and the 175-bp segment derived from IS*1006* differs from the IS*1008* sequence at 32 positions. The PCR products generated as described above (Table 4) that spanned the junctions of the 15 Ap^r^ Tp^r^ cointegrates formed between pRMH1013 (IS*1006*/*1008*) and R388::IS*1008* were sequenced. In all 15 instances, IS*1006/1008* had been distributed to the right-hand junction of the cointegrate, demonstrating that the reaction must have occurred between the right ends of IS*1008* and IS*1006/1008* (Fig. 4A). Conversely, in the 15 Ap^r^ Tp^r^ cointegrates formed via the reaction between pRMH1012 (IS*1008*) and R388::IS*1006/1008*, IS*1006/1008* had been distributed to the left-hand junction (Fig. 4C). The absence of the possible alternative configurations (Fig. 4B and D) indicates that the reaction could not occur via the left ends, where only 16 bp of sequence is shared. Taken together, these results indicate that the targeted conservative reaction can occur between two IS that share identical transposases and share nucleotide identity extending for a significant distance inward from one end.

To confirm this observation, we used a chemically synthesized IS*26* derivative designated IS*26*::*catA1*, which includes the *catA1* chloramphenicol resistance gene and an upstream promoter inserted downstream of the *tnp26* gene (Fig. 2B). In this construct, 775 bp at the left end and 44 bp at the right end are identical to IS2*6*. Hence, as the transposase produced is Tnp26, it should be able to pair productively with IS*26*, but in this case, the reaction should occur predominantly at the left end. Cointegrates formed between pRMH1015 containing IS*26*::*catA1* (Ap^r^ Cm^r^) and R388::IS*26* were detected at a mean frequency of 5.5 × 10^−4^ Ap^r^ Cm^r^ Tp^r^ cointegrates/Tp^r^ transconjugant (Table 3, last row), equivalent to the frequency for the reaction of IS*26* with IS*26* (Table 3). As determined by using the primers RH1451 with RH1472 and RH1452 with RH1471 described previously (6), which are specific for the position of IS*26* in R388 (but equivalent to those shown in Fig. 3A), 30 of 30 cointegrates examined (10 from each independent cross) had formed via a targeted reaction (Table 4). The sizes of the PCR products produced using the mapping primers revealed that, as predicted, in all cases the reaction occurred at the left end. The simplest explanation for these findings is that the targeted reaction requires progression of an HJ formed by the transposase (Tnp1008 or Tnp26).

### IS*1006* and IS*1006*/*1008* do not act together.

Although IS*1006* and the hybrid IS*1006/1008* also share an extended region of 175 bp of sequence identity, when pRMH1013 (IS*1006/1008*) was tested in combination with R388::IS*1006* or pRMH1011 (IS*1006*) with R388::IS*1006/1008*, Ap^r^ Tp^r^ cointegrates were formed at an averaged frequency of 5.6 × 10^−7^ or 6.9 × 10^−7^ per transconjugant (Table 3). PCR screening of 18 cointegrates showed that each had been formed via the untargeted route (Table 4), mediated by either IS*1006/1008* or IS*1006*. Hence, if the 175 bp of identical DNA sequence at the left ends of IS*1006* and IS*1006/1008* is sufficient to enable the targeted conservative reaction, then Tnp1006 and Tnp1008, after binding in *cis* to one end of the IS they were produced from, are unable to form a productive synapse.

To examine the if 175 bp of DNA identity is sufficient, we used IS2*6*-FS constructs, which include a frameshifting alteration, a duplication of 4 bp located at 114 to 117 from the left end in IS*26*-FS-L or a deletion of 13 bp located 184 bp from the right end in IS*26*-FS-R. These differences should severely impede progress of an HJ formed between IS*26* and either IS*26*-FS-L or IS*26*-FS-R. We have previously shown that when IS*26*-FS-L or IS*26*-FS-R was paired with IS*26*, cointegrates were formed at low frequency but always by the targeted route (6). The relatively even distribution of the IS*26*-FS to the left-hand or right-hand position in 10 cointegrates for each IS (6) indicates that 117 or 184 bp constituted sufficient stretches of identity to allow that IS end to be used. In this study, a further 10 cointegrates formed by pRMH962 containing IS*26*-FS-L with R388::IS26 or by pRMH762 containing IS*26* with R388::IS26-FS-R were isolated and examined as described previously (12). Again, approximately equal numbers of targeted cointegrates had arisen via a reaction at the left or via the right end. Totals from the two data sets were 13 on the left and 7 on the right for IS*26*-FS-R and 11 on the left and 9 on the right for IS*26*-FS-L.

As 117 bp was sufficient for the targeted reaction to occur, we conclude that the stretch of identity between IS*1006* and IS*1006/1008* of 175 bp should be sufficient. Hence, we conclude that the reason that IS*1006* did not work with IS*1006/1008* (Fig. 4E and F) arises from the differences in the catalytic domain of the Tnp1006 and Tnp1008 transposases. Though relatively few in number, 1 or more of these 16 differences appear to have been sufficient to impede formation of a productive synapse.

## DISCUSSION

**FIG 5:**
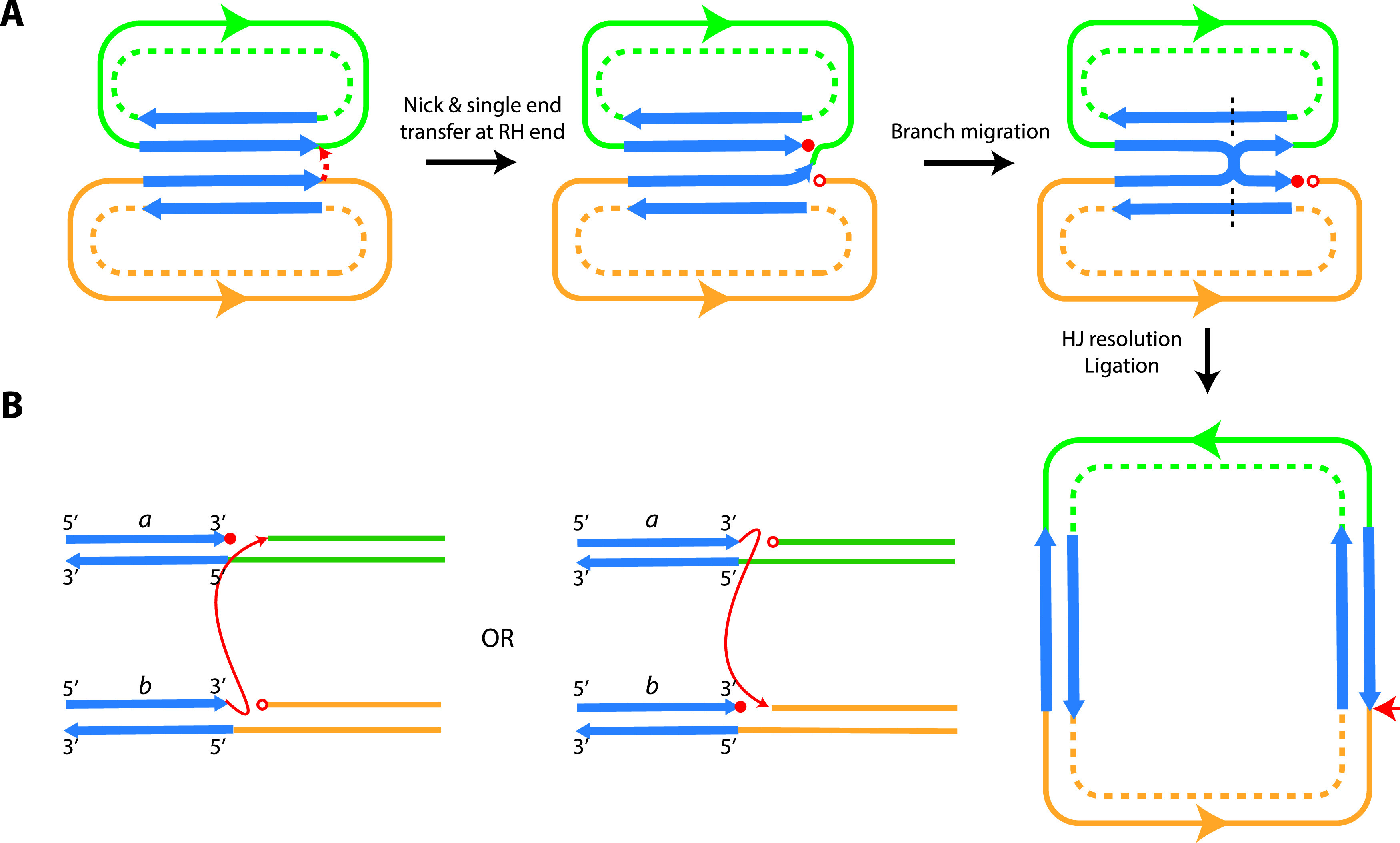
Holliday junction (HJ) branch migration model. (A) Single-ended strand cleavage and transfer during the targeted conservative reaction forming a nicked HJ, followed by ligation and HJ resolution to form a double-stranded cointegrate. The two participating molecules are indicated by green or orange lines, with the two DNA strands indicated by solid or dashed lines. The orientation of the molecules is indicated by a green or orange arrow. The two strands of the IS are indicated by blue arrows. Free 3′ or 5′ ends generated by strand cleavage and transfer are indicated by closed and open red circles, respectively. A dashed black line indicates HJ resolution, and the horizontal red arrow indicates the location of nicking and strand transfer. (B) The two possible intermediates formed via the reactions between the two right ends that lead to the same products are shown. The two other initial intermediates formed via reactions between the left ends are not shown. The two strands of the IS are indicated by blue arrows, with the strand direction indicated at the top, and the two IS are indicated by “*a*” or “*b*.” The different DNA contexts surrounding the IS are indicated by green or orange lines. Cleavage and strand transfer are indicated by a red arrow.

The work reported here increases to six the number of IS belonging to the IS*26* family, as recently defined (1), that have been shown experimentally to be able to form cointegrates using both copy-in and targeted conservative mechanisms. Investigations of the possible interactions between several pairs of closely related IS revealed that cointegrates formed via the targeted conservative route were recovered only when the same transposase was produced by both of the participating IS. However, there was an additional requirement for DNA identity at the IS end (left or right) involved in formation of a cointegrate. To explain the need for sequence identity at the reacting end, we propose that HJ branch migration is required to ensure that the single-end transfer catalyzed by the transposase leads to a cointegrate molecule. The model proposed is shown in Fig. 5A. Based on previous observations, we expect the transposase to bind preferentially in *cis* to one end of the IS it is produced from (7). We also expect that after the ends are brought together, presumably via formation of a transposase dimer, only a single-strand cleavage and strand transfer event occurs and this would form a nicked HJ. This is because when one strand transfer occurs it will cleave the phosphodiester bond that would be the target of a potential second strand transfer (Fig. 5B). As the ends involved in a conservative reaction are identical (left with left or right with right), strand cleavage and transfer can be initiated by one end of either participating IS and attack the equivalent end of the other IS. As the reaction can occur at either the left or the right end, there will be four initial intermediates in total (two are shown in Fig. 5B).

We have suggested previously that replication could be required to complete formation of the cointegrate (8). However, the observed requirement for extended DNA identity points to migration of the HJ. Supporting this conclusion, we have observed gene conversion occurring in the targeted conservative mode when one wild-type IS*26* has reacted with an IS*26* containing a mutation causing a single amino acid substitution located near the left end of the IS (C. H. Pong and R. M. Hall, unpublished observations), and this is consistent with the repair of mismatches arising from branch migration. When HJ migration is followed by HJ resolution involving the nontransferred strands, this would produce the cointegrate products observed (Fig. 5A). To the best of our knowledge, this mechanism has not been observed for any scenario involving a DDE transposase (12, 20) and hence represents a novel completely conservative reaction route.

Investigation of further details of this process is now needed. For example, if mutations were introduced into IS*1006/1008* in order to convert Tnp1008 produced to Tnp1006, pairing of this construct with IS*1006* would be predicted to move the reacting end to that derived from IS*1006* and pairing with IS*1008* would not produce products. Information on the extent of the DNA identity needed for an efficient reaction and what length of sequence identity is the minimum that can sustain the conservative reaction warrants further investigation. Whether the sequence identity must be within the IS or can also be adjacent to it also warrants investigation. The involvement of proteins that process the HJ, such as RecG or RuvABC, should shed further light on the mechanism.

Branch migration would stall when there are multiple mismatched nucleotides and particularly at clusters of mismatches, and this may be one reason why the closely related IS, such as IS*1006* and IS*1008* or IS*1006/1008*, were unable to act together in the targeted conservative reaction. However, IS*1006* and IS*1006/1008* could not interact to support conservative cointegrate formation even when extended sequence identity was available. This indicates that 1 or more of the modest number of amino acid differences between Tnp1008 and Tnp1006 (16 total, 6 conservative) are sufficient to prevent the reaction from occurring at all. Further work is needed to investigate this aspect. For example, the 16 mutants that each cause a single amino acid differences will need to be constructed and tested to begin to identify some of the key residues involved in multimerization and synapse formation.

## MATERIALS AND METHODS

### Bacterial strains and media.

E. coli DH5α (*supE44* Δ*lacU169* [ϕ80 *lacZ*ΔM15] *hsdR17 recA1 endA1 gyrA96 thi-1 relA1*) was used to propagate plasmids. E. coli UB5201 (*pro met recA* Nx^r^) was used as a donor in mating-out experiments, and E. coli UB1637 (*lys his trp lac recA* Sm^r^) was used as a recipient. Antibiotics (Sigma) were added at the indicated concentrations to either Mueller-Hinton broth or Mueller-Hinton agar, as appropriate: ampicillin (Ap), 100 μg/ml; nalidixic acid (Nx), 25 μg/ml; streptomycin (Sm), 25 μg/ml; and trimethoprim (Tp), 25 μg/ml.

### DNA manipulation.

Plasmid DNA was isolated by alkaline lysis as described previously (6). DNA was digested with restriction enzymes according to the manufacturer’s instructions, and fragments were separated through 0.7% agarose in 1× Tris-acetate-EDTA (TAE) buffer. The size standards were a 1-kb ladder and λ-HindIII (New England BioLabs). PCRs were performed using conditions previously described (6), and routine sequencing of PCR products was performed as previously described (6). The sequences for all primers used in this study are listed in Table S1.

### Plasmid construction.

The plasmids used in this study are listed in Table 1. Gibson Assembly (New England BioLabs, USA) was used to generate pRMH1011, pRMH1012, pRMH1013, R388::IS*1006*, R388:IS*1008*, R388::IS*1006*/*1008*, and R388::IS*26*-2 using the primers listed in Table S1 with standard manufacturer conditions. Inserts were cloned into the BamHI site of pUC19 or between the HindIII sites of the conjugative IncW plasmid R388. Each insert contained the IS of interest, plus approximately 100 bp of flanking DNA (except for IS*1008*, where about 500 bp is included on one side). DNA from pD36-4 (21) (GenBank accession no. CP012956) was used as the template for IS*1006* (bases 39531 to 40583 were amplified for cloning), pA297-3 (16) (GenBank accession no. KU744946) DNA was used as the template for IS*1008* (bases 88655 to 90375 were amplified for cloning), and pJ9-3 (bases 42241 to 43233; GenBank accession no. CP041590) was used as the template for IS*1006*/*1008*. All clones were transformed into chemically competent E. coli DH5α cells by heat shock. The presence of the insert in pUC19 was confirmed by amplification of the insert followed by sequencing using the pUC19 universal primers. Likewise, primers RH2735 and RH2563 were used to amplify and sequence the insert in R388. The synthetic IS*26*::*catA1* was created with flanking sequences identical to those in pRMH977 and cloned into the BamHI site of pUC19 via Gibson assembly to form pRMH1015.

### Mating-out cointegration assays.

Donors for cointegration assays were generated via conjugation of either R388 (Su^r^ Tp^r^) or an R388 derivative containing the IS of interest into E. coli UB5201 (*recA* negative; Nx^r^) cells containing nonconjugative pUC19-derived plasmids containing IS*26* (pRMH977; Ap^r^), IS*1006* (pRMH1011; Ap^r^), IS*1008* (pRMH1012; Ap^r^), or IS1006/1008 (pRMH1013; Ap^r^). Cointegrate formation was assessed by mating these strains with UB1637 (*recA* negative; Sm^r^) and selecting for Ap^r^ Sm^r^ Tp^r^ colonies. pRMH977 (IS*26*) and R388::IS*26*, as previously tested (6, 8, 9), were included as a comparison. The transposition frequency was calculated as the number of Ap^r^ Sm^r^ Tp^r^ transconjugants (cointegrates) per Tp^r^ Sm^r^ transconjugant (R388 or R388 derivative).

### Restriction mapping of untargeted cointegrates.

IS*1006* and IS*1008* each contain a single BglII restriction site, though the site is in a different position in each IS. The mapping strategy used to determine the cointegrate boundaries utilized this site combined with two BglII and two BsiWI sites in the R388 backbone. Briefly, to determine the positions of cointegrate junctions in the R388 backbone, plasmid DNA was recovered from independent cointegrates and digested with BglII or BsiWI or both, and the restriction fragments were separated in a 0.7% TAE agarose gel. The BglII/BsiWI double digestion of R388 produces four fragments of 14.5 kb, 10.6 kb, 6.8 kb, and 1.9 kb. If a cointegrate has formed, the presence of the BglII site in the IS at each boundary of the fusion splits one of the expected R388 fragments in two and forms an additional fragment corresponding to the original pUC19-based plasmid.

### PCR mapping of targeted cointegrates.

Targeted conservative cointegrate formation was detected by PCR across each IS in the predicted cointegrate. Primers RH2563 and RH2735 in R388 (Table S1), flanking the R388 HindIII sites, were used in combination with the pUC19 universal primers (Table S1). When targeted cointegrates were not detected by PCR, primers RH2563 and RH2735 and the pUC19 universal primers were used to confirm whether one of the original IS in the reaction was still located in the same position, i.e., had not formed a cointegrate (Fig. 3).
