# Supplementary material for: Targeted Conservative Cointegrate Formation Mediated by IS26 Family Members Requires Sequence Identity at the Reacting End
Source: mSphere. 2021 Jan 27;6(1):e01321-20. doi: 10.1128/mSphere.01321-20 (PMC7885326; doi:10.1128/mSphere.01321-20)
Supplement: TABLE S1 [file mSphere.01321-20_st001.docx]

Table S1. Primers used in this study.

| Primer | Target | Sequence (5′ – 3′)^a^ |
| --- | --- | --- |
| *Gibson cloning into pUC19 BamHI site* | |  |
| RH2716 | IS*1006* in pD36-4 | **ATTCGAGCTCGGTACCCGGG**CGTTTCCTGCCCGCTGATTG |
| RH2717 | IS*1006* in pD36-4 | **CCTGCAGGTCGACTCTAGAG**AGGGAATGCACCATGACAC |
| RH2596 | IS*1008* in pA297-3 | **ATTCGAGCTCGGTACCCGGG**CGCTTCAGCATTTTGTTTTG |
| RH2598 | IS*1008* in pA297-3 | **CCTGCAGGTCGACTCTAGAG**GGTATTGGTCTGGCAGTCGT |
| RH2750 | IS*1006*/1008 in pJ9-3 | **ATTCGAGCTCGGTACCCGGG**GGCTCAAGAATTTGCCAGAG |
| RH3001 | IS*1006*/1008 in pJ9-3 | **CCTGCAGGTCGACTCTAGAG**CTGTTTTCGACATCCCGT |
|  |  |  |
| *Gibson cloning into R388 HindIII site* | |  |
| RH2744 | IS*1006* in pD36-4 | **AGCGAGGGCTTTACTA**CGTTTCCTGCCCGCTGATTG |
| RH2745 | IS*1006* in pD36-4 | **AAGCCCCACGCATCA**AGGGAATGCACCATGACAC |
| RH2746 | IS*1008* in pA297-3 | **AGCGAGGGCTTTACTA**CGCTTCAGCATTTTGTTTTG |
| RH2747 | IS*1008* in pA297-3 | **AAGCCCCACGCATCA**GGTATTGGTCTGGCAGTCGT |
| RH2748 | IS*1006*/1008 in pJ9-3 | **AGCGAGGGCTTTACTA**GGCTCAAGAATTTGCCAGAG |
| RH2749 | IS*1006*/1008 in pJ9-3 | **AAGCCCCACGCATCA**CTGTTTTCGACATCCCGT |
|  |  |  |
| *Screening primers* | |  |
| RH2735 | Upstream of R388 HindIII site | GCCCTACACAAATTGGGAGA |
| RH2563 | Downstream of R388 HindIII site | GCAATTATGAGCCCCATACC |
| RH2702 | pUC19 universal forward | GTTGTAAAACGACGGCCAGT |
| RH2703 | pUC19 universal reverse | CACAGGAAACAGCTATGACC |
| RH1471 | IS*26* in R388::IS*26* | CCGCTCCAAAAACTATCCAC |
| RH1472 | IS*26* in R388::IS*26* | ATCGGAAATGGTTGTGAAGC |
|  |  |  |
| *Cointegrate mapping primers* | |  |
| RH3004 | R388-IS1006 Coint 1 | GCTGACCCAAGACACCTTTT |
| RH3005 | R388-IS1006 Coint 1 | CCGCTATTGCTCCAAAAATC |
| RH3006 | R388-IS1006 Coint 2 | TGTGGTGTAACGCGGTTCTA |
| RH3007 | R388-IS1006 Coint 2 | CGGCTACCTCATTCGAGAAG |
| RH3008 | R388-IS1006 Coint 3 | TATTGTCGAAACGCTGAACG |
| RH3009 | R388-IS1006 Coint 3 | AATGCGTCAGCCAAAGAATC |
| RH3010 | R388-IS1006 Coint 4 | CATTCTTGCGAATGAGACGA |
| RH3011 | R388-IS1006 Coint 4 | CATCCTAATCGGCCATCACT |
| RH3012 | R388-IS1006 Coint 5 | TGCTTTGATTTCCGAGTGAA |
| RH3013 | R388-IS1006 Coint 5 | AACGACGAATGACGAGCAG |
| RH3014 | R388-IS1006 Coint 6 | TGGCGTGGTTTCTAGCTCTT |
| RH3015 | R388-IS1006 Coint 6 | CACGATGGAGGAAAGACCAT |
| RH3016 | R388-IS1008 Coint 1 | GATCATTTGCTTGGCGGTAT |
| RH3017 | R388-IS1008 Coint 1 | TCTTGCAGATCGAACGTGAC |
| RH3018 | R388-IS1008 Coint 2 | CGTTGGTCATGTGTGACTCC |
| RH3019 | R388-IS1008 Coint 2 | TGAAACGCTGCGATCTATTG |
| RH3020 | R388-IS1008 Coint 3 | CGGCTTCTTTCCTTTGGAAG |
| RH3021 | R388-IS1008 Coint 3 | ACCGGCGAAGAAAAGAAAAT |
| RH3022 | R388-IS1008 Coint 4 | TCAAGACGGCTGTGAGATTG |
| RH3023 | R388-IS1008 Coint 4 | GGAAGCGGTAGCTTCACAAC |
| RH3024 | R388-IS1008 Coint 5 | CGTGATGCCTGCTTGTTCTA |
| RH3025 | R388-IS1008 Coint 5 | GCCTTGCTGTTCTTCTACGG |
| RH3026 | R388-IS1008 Coint 6 | TGATATCGACGAGGTTGTGC |
| RH3027 | R388-IS1008 Coint 6 | CGGACTGCAAGTGATCTTGA |
| RH3028 | R388-IS1006/1008 Coint 1 | AACCGTATTGCTCGACTGCT |
| RH3029 | R388-IS1006/1008 Coint 1 | CGAACGCTTCACGACTGTTA |
| RH3030 | R388-IS1006/1008 Coint 2 | CGGTTCTACAGCCGAGAAAG |
| RH3031 | R388-IS1006/1008 Coint 2 | TAGTCGTAGGCTTCGCGATT |
| RH3032 | R388-IS1006/1008 Coint 3 | AACAAGGGCTTGAAGCGATA |
| RH3033 | R388-IS1006/1008 Coint 3 | CCGAAAACCAATGAGGACAT |
| RH3034 | R388-IS1006/1008 Coint 4 | CAAGGGTGGTTGACAGGTCT |
| RH3035 | R388-IS1006/1008 Coint 4 | GGAGTCACACATGACCAACG |
| RH3036 | R388-IS1006/1008 Coint 5 | CATTCTTGCGAATGAGACGA |
| RH3037 | R388-IS1006/1008 Coint 5 | ACATGGCCGAAATTGAGAAC |
| RH3038 | R388-IS1006/1008 Coint 6 | CATGAGAAGCGCGTCATAAA |
| RH3039 | R388-IS1006/1008 Coint 6 | CGACGTCTCTACGACGATGA |

^a^ Sequence in bold denotes the pUC19- or R388-specific portion of a Gibson primer, and the underlined sequence denotes the insert-specific portion.
